# Medical History from the Earliest Times

**Published:** 1893-03-11

**Authors:** E. T. Withington


### Medical History from the Earliest Times.

By E. T. Withington, M.A., M.B. Oxon.

### XLVI.—THE MYSTICS, SYMPATHY, AND SIGNATURES.

The revival of Greek learning and the discovery of the New World at the close of the fifteenth century produced an intellectual ferment in which the minds of men were singularly attracted by all things new and extravagant; and throughout the sixteenth century we find distinguished men devoting themselves to the serious study of such works as the Jewish Cabbala, and the forged theosophic fragments attributed to Orpheus, Pythagoras, and Hermes Trismegistus. The results of this mysticism were manifested in general history by what has been well called the "witch mania," and in that of medicine by the extravagances of the astrologers and alchemists, and it is to the credit of the "Greeks," or "Hippocratic physicians" as they called themselves, that they vigorously opposed all three.

Passing over Dr. John Weyer and his crusade against the witch hunters, we may gather some interesting details as to the astrological doctors from the Medical Epistles of John Lange, a pupil of Nicholas Leonicenus, and physician to four Electors Palatine (1485-1565). The doctrine that the heavenly bodies, and particularly the signs of the zodiac, exert an influence on the human frame, was held throughout the middle ages, and was especially favoured by the Arabist school of medicine; but it received its most extraordinary development from the mystics of the sixteenth century. Astrological calendars were compiled to show the proper days and seasons in which alone medicine could be given or venæsection performed, and these were sometimes confirmed by the decrees of town councils, as at Bruges, where the barbers were forbidden to shave or bleed persons on the days marked dangerous in the "great and perpetual almanack" of Bruhesen (1550). Lange tells us that some of these calendars forbade any medicines to be given when the moon was in the signs of the ram, the bull, or the he-goat, for they are ruminating animals, and according to Hermes Trismegistus, all medicine given at such times tends to return to the mouth. He adds that there was a tragic as well as a comic side to the superstition, "for it was thus that that promising youth, Peter Lemberg, lost his life. He was seized with an acute pleurisy, and the doctor recommended bleeding. Then came a runaway monk with an astrological calendar, who said the physician knew nothing, and that the patient could not be bled for three days, when the moon would be in the sign of the fishes. He died, however, the following evening. "O vanitas vanitatum, et super omnia astrologorum vanitas!"

The chemical mystics, as they may be called, form a much more important class, and their influence on the healing art was far from being entirely unfavourable. They are represented in medical history by Basil Valentine, Paracelsus, and Van Helmont, men whose relative fame is perhaps in inverse proportion to their actual merit. Of the first, we know only what can be gathered from his works, which were not published till a century after his death. He tells us he was a Benedictine monk, and legend connects him with the monastery at Erfurt, but no list of the order contains his name, which is probably an assumed one. Finding that the rule of St. Benedict still left leisure for the intrusion of idle thoughts, he began to study the secrets of Nature, in which he was much aided by ancient books contained in the monastery library. A brother monk had long suffered from a disease which baffled all physicians. In vain did Basil investigate the "anatomy" and extract the "quintessences" of numberless herbs during six years, till at last he turned to minerals, and found one of many colours, the "spirit," of which sufficed to cure the monk in a few days. For the rest of his life the grateful patient devoted an hour daily to special prayer for Basil, who, as the result of these and his own petitions, discovered many things hidden from those who think themselves wise and prudent. Nor is this boast unjustified, for the works of Basil Valentine contain the earliest mention of zinc and bismuth, of hydrochloric acid, sugar of lead, nitrate of mercury, and numerous salts of antimony, besides the modern methods of producing ammonia and sulphuric acid. By distilling this last substance with alcohol he obtained a pleasantly smelling liquid, which must have been ether, and which he recommends in many diseases. To Basil the great end of alchemy is the discovery of new remedies. Most chemists, he says, strive only after riches, caring nothing for their fellows, and thereby heap up judgment against themselves at the last day, but it would ill beseem a member of his sacred order to follow such examples, and his only object is to impart the knowledge of useful medicines to those who come after him. Mercury contains the highest arcanum of human health, and its spirit makes men and animals young like the eagle. The spirit of quicklime dissolves stone, and is a sovereign cure for gout. Valuable remedies may be got from arsenic, nitre, copper, and lead, but the highest and noblest of drugs is the quintessence of antimony, which was specially created for the purification of mankind, and not only cures diseases, but rejoices the heart and increases chastity and piety. The physicians of his day, adds Basil, avoid chemistry because they are afraid of dirtying their hands and raising the price of soap, and he proceeds to attack them in language closely resembling that of Paracelsus, though somewhat less vigorous, "for I am a spiritual man, and bound by the rule of my order to have patience with all, or I could speak much more strongly." Had Basil's works been published when they were written, he would have been the founder of medical chemistry, an honour which has been usurped by Paracelsus, whose writings contain so exact a reproduction of Basil's theories, discoveries, and even language that it is impossible to doubt that he had seen his predecessor's works in manuscript, and made extensive use of them.

